# Pneumothorax detection in chest radiographs: optimizing artificial intelligence system for accuracy and confounding bias reduction using in-image annotations in algorithm training

**DOI:** 10.1007/s00330-021-07833-w

**Published:** 2021-03-27

**Authors:** Johannes Rueckel, Christian Huemmer, Andreas Fieselmann, Florin-Cristian Ghesu, Awais Mansoor, Balthasar Schachtner, Philipp Wesp, Lena Trappmann, Basel Munawwar, Jens Ricke, Michael Ingrisch, Bastian O. Sabel

**Affiliations:** 1 Department of Radiology, University Hospital, LMU Munich, Marchioninistr. 15, 81377 Munich, Germany; 2 X-Ray Products, Siemens Healthineers, Forchheim, Germany; 3 Digital Technology and Innovation, Siemens Healthineers, Princeton, NJ USA; 4 Comprehensive Pneumology Center (CPC-M), Member of the German Center for Lung Research (DZL), Munich, Germany

**Keywords:** Artificial intelligence, Chest radiography, Pneumothorax, Chest tubes

## Abstract

**Objectives:**

Diagnostic accuracy of artificial intelligence (AI) pneumothorax (PTX) detection in chest radiographs (CXR) is limited by the noisy annotation quality of public training data and confounding thoracic tubes (TT). We hypothesize that in-image annotations of the dehiscent visceral pleura for algorithm training boosts algorithm’s performance and suppresses confounders.

**Methods:**

Our single-center evaluation cohort of 3062 supine CXRs includes 760 PTX-positive cases with radiological annotations of PTX size and inserted TTs. Three step-by-step improved algorithms (differing in algorithm architecture, training data from public datasets/clinical sites, and in-image annotations included in algorithm training) were characterized by area under the receiver operating characteristics (AUROC) in detailed subgroup analyses and referenced to the well-established “CheXNet” algorithm.

**Results:**

Performances of established algorithms exclusively trained on publicly available data without in-image annotations are limited to AUROCs of 0.778 and strongly biased towards TTs that can completely eliminate algorithm’s discriminative power in individual subgroups. Contrarily, our final “algorithm 2” which was trained on a lower number of images but additionally with in-image annotations of the dehiscent pleura achieved an overall AUROC of 0.877 for unilateral PTX detection with a significantly reduced TT-related confounding bias.

**Conclusions:**

We demonstrated strong limitations of an established PTX-detecting AI algorithm that can be significantly reduced by designing an AI system capable of learning to both classify and localize PTX. Our results are aimed at drawing attention to the necessity of high-quality in-image localization in training data to reduce the risks of unintentionally biasing the training process of pathology-detecting AI algorithms.

**Key Points:**

*• Established pneumothorax-detecting artificial intelligence algorithms trained on public training data are strongly limited and biased by confounding thoracic tubes.*

*• We used high-quality in-image annotated training data to effectively boost algorithm performance and suppress the impact of confounding thoracic tubes.*

*• Based on our results, we hypothesize that even hidden confounders might be effectively addressed by in-image annotations of pathology-related image features.*

**Supplementary Information:**

The online version contains supplementary material available at 10.1007/s00330-021-07833-w.

## Introduction

Chest radiography is the most commonly performed diagnostic imaging procedure throughout the world and therefore has a relevant impact on public health [1, 2]. Pneumothorax (PTX) is a potentially life-threatening pulmonary disorder and therefore needs to be reliably and time-critically detected. Treatment options for PTX may include observation, thoracic tube (TT) insertion, or surgery [3–7]. A PTX is usually detected by chest radiography. However, large volumes of chest radiographs (CXR) in routine clinical environment may yield longer turnaround times for radiology reporting which can delay urgent treatment; this issue as well as latent critical findings can be potentially addressed by the use of artificial intelligence (AI)–assisted reporting or an AI-based image triage. Several AI algorithms, trained on publicly available datasets, have demonstrated potential to detect PTX in CXRs with diagnostic accuracies that have been quantified by area under receiver operating characteristics (AUROCs) of up to 0.937 [8–13]. In studies evaluating these algorithms, the performance was evaluated on data derived from public datasets [8, 14, 15]. However, limited labeling within these datasets does not allow a detailed subgroup analysis or the identification of confounders and their impact on the performance of AI algorithms.

Based on a benchmarking cohort of 6434 supine chest radiographs (SCXR) radiologically annotated for PTX size, location, and inserted TTs, a previous study [16] identified TTs to be relevant confounders that can potentially eliminate the discriminative power of PTX-detecting algorithms trained on publicly available datasets without in-image annotations (ChestX-ray14 and the dataset derived from the prostate, lung, colorectal, and ovarian cancer screening trial [PLCO]) [15, 17].

Here, we hypothesize that in-image pixel annotations of the dehiscent visceral pleura, as well as a rigorous algorithm design that enables it to effectively learn from this information, lead to a large increase in overall performance and significantly reduce the confounding bias caused by inserted TTs. These improvements are essential to bring PTX detection algorithms to clinical routine by offering support for clinical decision-making, reducing the number of missed findings, and improving the turnaround time for radiology reporting; the latter one was quantified based on our study cohort. Furthermore, our experiments might also demonstrate that in-image pathology annotations in general is a promising technique to mitigate biases (possibly unknown) induced due to confounding imaging features in algorithm training.

## Materials and methods

Approval of the institutional ethics commission was obtained for this study (approval number 19-541).

### Patient identification and image annotation

**Fig. 1:**
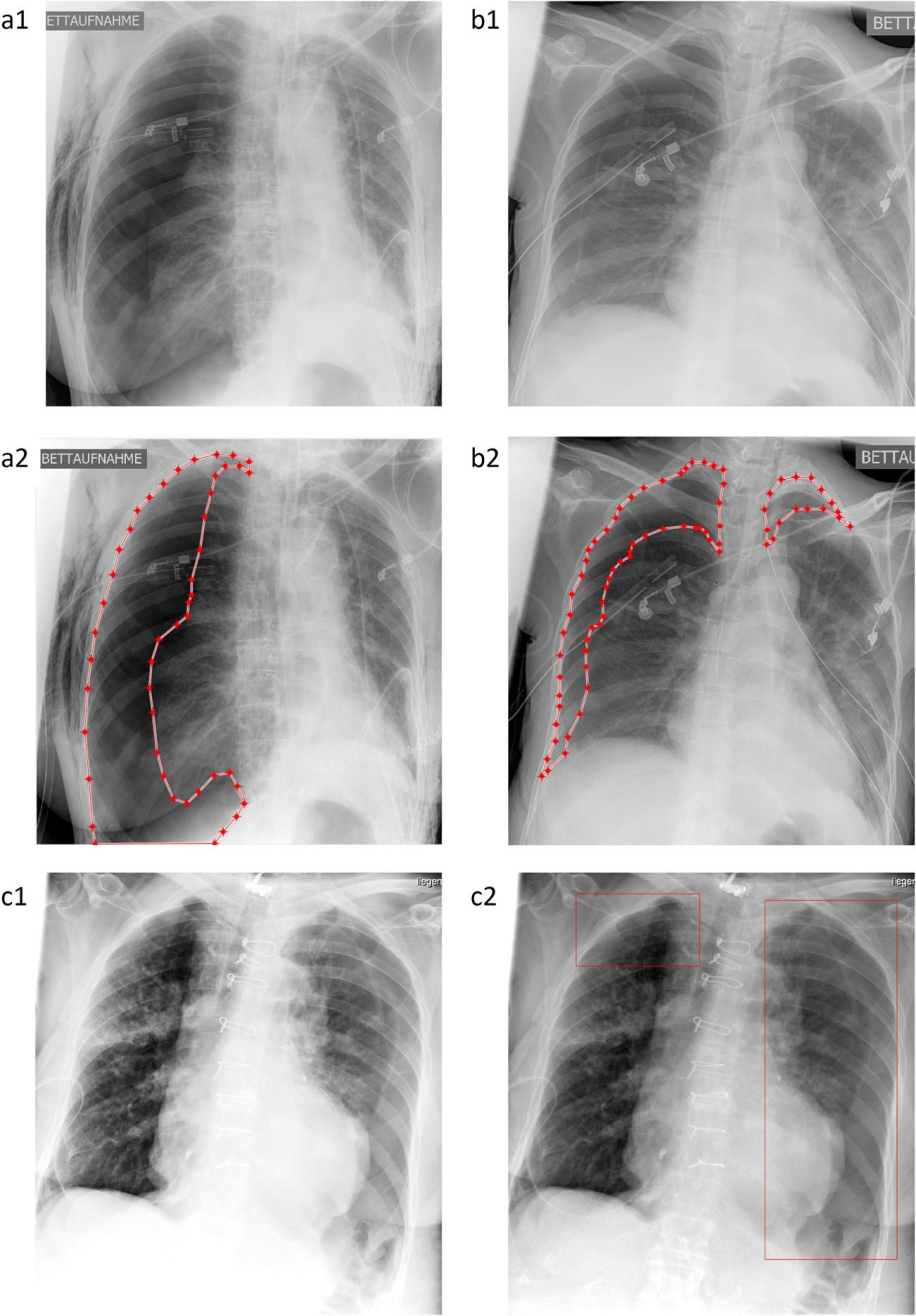
Annotation of the dehiscent visceral pleura for algorithm training (**a**, **b**) and resulting localization of algorithm findings (“Algorithm 2”, **c**). (**a**, **b**): The SCXRs shows a unilateral (**a1**) / bilateral (**b1**) pneumothorax. Pixel coordinates of the dehiscent visceral pleural and thoracic wall are annotated and connected to a polygon representing the pneumothorax shape (**a2**, **b2**). (**c1, c2**): “Algorithm 2” allows for the dedicated localization of the image features yielding the algorithm score representing the algorithm confidence for a PTX (the same SCXR is illustrated as original contrast-enhanced DICOM (**c1**) and AI-finding-enriched image (**c2**))

Patients were retrospectively identified by data research (different search criteria based on radiology reports from 2010 to 2018 to separately identify PTX-positive/negative images, consequently clinically non-consecutive cohort with a targeted PTX overall prevalence of approx. 25%) in our institutional Picture Archiving and Communication System (PACS). We exclusively focused on supine chest radiographs due to the more challenging image interpretation and more frequently inserted thoracic tubes. The comparison of time stamps of our PACS corresponding to image acquisition and radiology reporting allowed us to measure the mean radiology reporting time. DICOM images of the identified cases were exported and manually checked for the existence of PTXs during image annotation as described below. Inconclusive cases with questionable PTXs (e.g., very small PTXs) were handled based on their plausibility through prior medical history and imaging.^1^ No other exclusion criteria have been applied so that we expect only PTX-related variations from a clinically representative routine cohort. Consequently, we identified 1526 PTX-positive images (1066 different patients) and 4587 PTX-negative images (3294 different patients) from adult patients (age older than 21) of the benchmarking cohort previously introduced by Rueckel et al [16]. Age (PTX-positive cases: 60 ± 16 years, PTX-negative cases: 66 ± 15 years) and gender (PTX-positive cases: 45.0% female, PTX-negative cases: 40.1% female) were recorded. Data was directly extracted from clinical routine without applying any quality-related exclusion criteria; therefore, data also includes examinations of limited quality (e.g., oblique projection, overexposure, or limited inspiration depth). PTX size (maximum interpleural space < 1cm/1–2 cm/> 2 cm), PTX location (affected side), and the presence of inserted TTs were qualitatively annotated allowing for subgroup definitions. Subgroups based on PTX size and inserted TTs have been built. Fifty percent of each subgroup’s images have been considered for algorithm training (see Table 2). The remaining images were assigned to the evaluation dataset (see Table 1) and have never been used for algorithm training or optimization. The PTX-positive images used for algorithm training (algorithm 2, see below) were annotated using an internal software tool (allowing window and zoom) with polygons defining the PTX shape (see Fig. 1a2/b2). Annotations have been carried out by two well-trained fourth-year medical students (directly supervised annotation for the first approx. 10–50 images, in the further course annotation review of questionable cases by a radiology resident with 3 years of experience in thoracic imaging) and radiology experts. All in-image pixel annotations of PTX-positive image data used for algorithm training were verified by expert radiologists.

**Table 1:** Study Cohort Subgroup Characteristics for Algorithm Evaluation. PTX-positive cases are radiologically annotated for PTX size, PTX location (unilateral vs bilateral) and inserted thoracic tubes. PTX-negative control cases are radiologically annotated for inserted TTs

Unilateral PTX (*n* = 677)	Thoracic tube		Sum/fraction [*n*/%]
	Yes [*n*]	No [*n*]	
Dehiscence < 1 cm	203	69	272/40.2%
Dehiscence 1–2 cm	162	46	208/30.7%
Dehiscence > 2 cm	142	55	197/29.1%
Sum/fraction [*n*/%]	507/74.9%	170/25.1%	
Bilateral PTX (*n* = 83)	Thoracic tube		Sum/fraction [*n*/%]
	Yes [*n*]	No [*n*]	
Max. dehiscence < 1 cm	17	3	20/24.1%
Max. dehiscence 1–2 cm	29	1	30/36.1%
Max. dehiscence > 2 cm	28	5	33/39.8%
Sum/fraction [*n*/%]	74/ 89.2%	9/10.8%	
Control cases (*n* = 2302)	Thoracic tube		Sum [*n*]
	Yes [*n*]	No [*n*]	
PTX-negative	293	2009	2302
Fraction [%]	12.7%	87.3%

Furthermore, additional training dataset was constructed including cases from various clinical sites^2^ as well as data from the ChestX-ray14 dataset [17] and the Society of Imaging and Informatics (SIIM) PTX challenge. For all positive cases in the training dataset, the in-image annotations of the dehiscent visceral pleura were produced (for the SIIM challenge data, such annotations were already available). These annotations have been verified and corrected by expert radiologists. All PTX-negative cases have been randomly selected from the ChestX-ray14 [17] and various clinical sites. The selection was performed with a natural language processing (NLP) system that has parsed available radiology reports.

### Artificial intelligence algorithms

CheXNet is an algorithm trained and validated on the ChestX-ray14 dataset [17] and originally introduced by Rajpurkar et al [18] with AUROCs for PTX detection up to 0.8887. It outperformed recent AI solutions trained on comparable public training data and therefore is often used as baseline method [9, 14, 17–19]. Python implementation of the algorithm is available on GitHub, provided by Weng et al (https://github.com/arnoweng/CheXNet) [19].

Algorithm 0 is an internal prototype (intermediate version), based on a deep learning solution trained on the ChestX-ray14 and PLCO datasets [15, 17]. A detailed description of the method is available in Guendel et al [20, 21] and provided as supplemental file. Please note that Algorithm 0 was designed to simultaneously classify various abnormalities (including PTX) from frontal chest radiographs. Optimization of the system was performed such that the optimal average AUROC performance is achieved. There was no focus on pneumothorax only during the training and optimization routine.

Algorithm 1 is an internal prototype (intermediate version), based on a novel deep learning solution that is designed to both classify and localize pneumothoraxes in chest radiographs. The algorithm uses a new hybrid learning model that can learn from image-level binary labels indicating the presence of pneumothorax, and from detailed contoured annotations of the dehiscent visceral pleura. The algorithm comprises two main components, namely, dehiscent visceral pleura localization module and pneumothorax classification module. The localization module is an encoder-decoder convolutional architecture estimating the contours of dehiscent visceral pleura as a binary image mask. This is followed by the classification module to obtain an image-level probability score for pneumothorax. Briefly, the architecture of the classification module is inspired by the DenseNet architecture [22]; however, further details about the architecture can be found in the Supplemental Material. Algorithm 1 was trained on the ChestX-ray14 dataset [17] with NLP image labels for both positive/negative PTX cases and a set of 2349 positive PTX cases (from the SIIM PTX challenge) with in-image annotation.

Algorithm 2 is an internal prototype (final version), based on a similar deep learning architecture as Algorithm 1. The difference is that, unlike Algorithm 1, the training data set of Algorithm 2 also included images from various clinical sites^2^. Please recall that, in this dataset, all positive cases have been annotated with precise in-image annotations of the dehiscent visceral pleura. We did not use positive PTX cases based on NLP labeling. The negative cases have been sampled randomly from a large cohort of data from different clinics as well as public data using NLP; the number of randomly selected negative PTX cases optimally balanced maximized system performance with training duration.

A detailed description of learning models used in Algorithms 0, 1, and 2 is provided as Supplemental Material. Table 2 provides an overview of the algorithms.

**Table 2:** Overview of the AI algorithms reviewed in our study. We consider four algorithms for evaluation: the CheXNet algorithm introduced by Rajpurkar et al (1) and three internal prototypes: “Algorithm 0,” “Algorithm 1,” and “Algorithm 2.” While CheXNet and “Algorithm 0” are trained on positive PTX cases solely identified through NLP, some positive cases (“Algorithm 1”)/all positive PTX cases (“Algorithm 2”) used for the training were annotated with a contour of the dehiscent visceral pleura. These in-image annotations have been defined/verified by expert radiologists. Training data from the benchmarking clinical site included positive as well as negative PTX cases, each with as well as without inserted TTs (^1^). The SIIM provides segmentation of the pleural line, while the original ChestX-ray14 dataset provides only image labels. When we say SIIM, we use 2349 images with annotation of pleural line. All these images come from the ChestX-ray14 data. “Algorithm 1” and “Algorithm 2” provide not only an image-level probability for pneumothorax (like CheXNet and “Algorithm 0”) but also instance level bounding box detections of the affected region

Method	Status	Functionality	Training data			
			Dataset (fraction [%])	Cases [sum, #] [PTX+, #] [PTX+, %]	Shape annotation [% of PTX+ cases]	From same clinical site as test data [%]
CheXNet	Publicly available	Classification only	ChestX-ray14 [17] (100%)	112,120 2793 2.49%	0%	0%
Algorithm 0	Intermediate version	Classification only	ChestX-ray14 [17] (38%) PLCO [15, 17] (62%)	297,541 4992 1.67%	0%	0%
Algorithm 1	Intermediate version	Classification and localization	ChestX-ray14 [17], SIIM^2^ (100%)	112,120 4992 4.45%	47%	0%
Algorithm 2	Final version	Classification and localization	ChestX-ray14 [17], SIIM^2^ (42%) Various clinical sites (58%)^2^	75,067 3993 5.31%	100%	3.38%^1^

### Image analysis, result quantification, and statistics

Exported SCXR DICOMs were analyzed by the previously described algorithm prototypes that had been installed on separate research computers. Algorithms produced uncalibrated classifier scores between 0 and 1, representing algorithm’s confidence for existing PTX in the SCXR.

The performance of the AI algorithms was quantified using receiver operating characteristic (ROC) analysis based on the subgroups differing in PTX size (for size thresholds, see above), PTX location, and inserted TTs in PTX-positive cases and/or PTX-negative controls. Subgroup analysis of bilateral PTX cases was based only on PTX size on the predominant side (a very small number of images without any TTs inserted did not allow for subgroups differing in the presence of TTs). Subgroup ROC analysis including the calculation of the AUROC, the quantifying algorithm’s discriminative power, and graphic illustrations was semi-automatically performed by R-Studio (Version 1.2.5001, RStudio Inc., Boston, USA). Here, it has to be kept in mind that AUROCs are known to be independent from underlying pathology prevalence [23, 24]. For that reason, the partially overrepresented fraction of PTX-positive images (overall prevalence approx. 25% higher than clinically expected, considered subgroups with much lower prevalences) should not bias the quantified algorithm performances and also the comparison of subgroups of different PTX prevalences is possible. Significance analysis was based on AUROC’s 95% confidence intervals (95% CI) and ROC curves have been compared according to Delong et al and Sun et al (R-package roc.test) [25, 26]. GraphPad Prism (Version 8, GraphPad Software, San Diego, USA) was additionally used for graphical illustrations.

## Results

The mean radiology reporting time, without AI assistance, was measured to be 01:30 h (PTX-positive SCXRs)/1:40 h (PTX-negative SCXRs) within our study cohort. This delay compared with an estimated isolated reading time of 1–2 min per SCXR for experienced radiologists emphasizes the potential added clinical value of an AI-based preselection of images for a prioritized reporting. Radiology reporting turnaround times of images with significant findings could be significantly reduced in such an AI-based image triage approach.

**Fig. 2:**
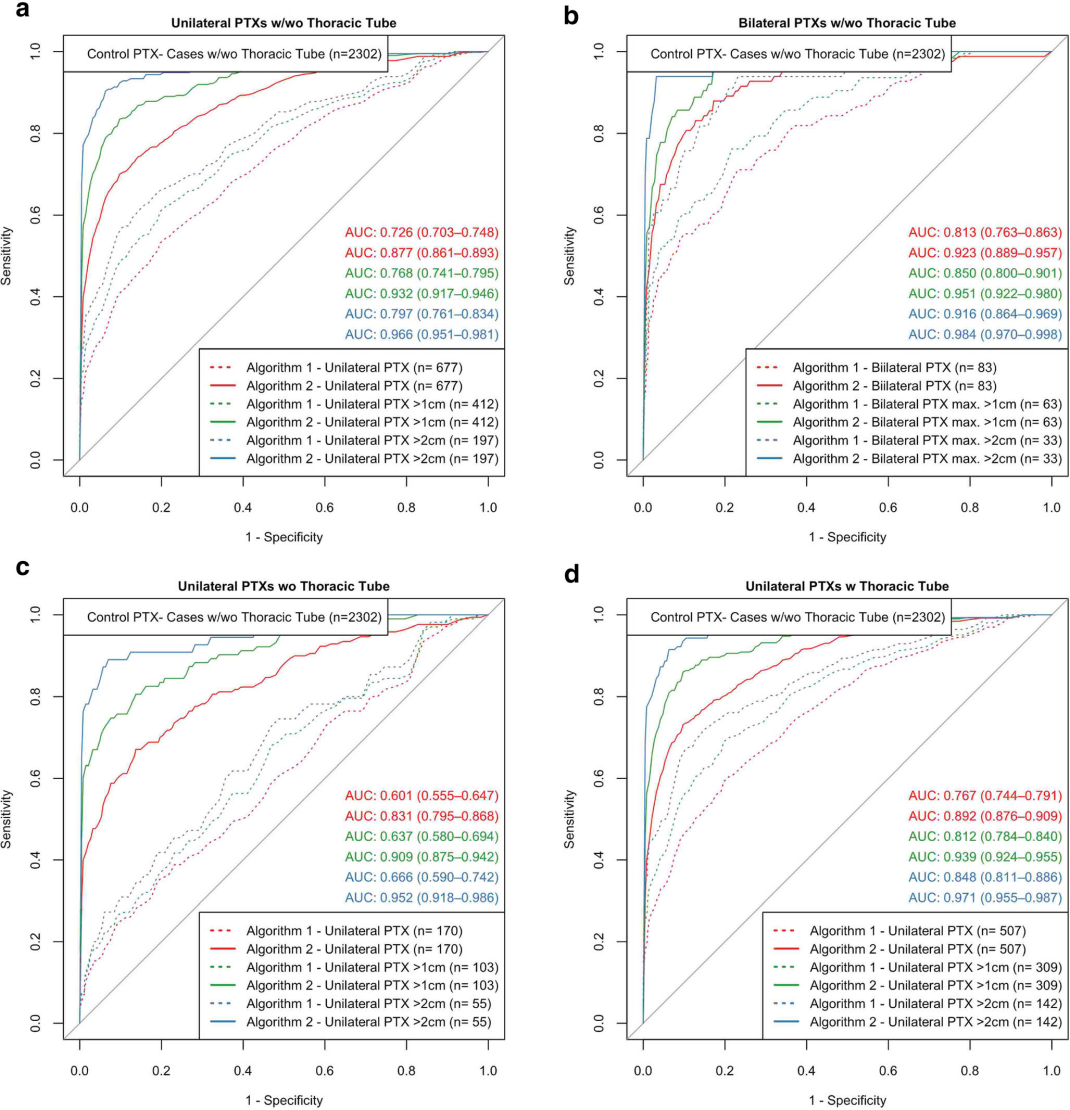
Algorithm discriminative power in pooled subgroups (“Algorithm 1,” “Algorithm 2”). (**a**): “Algorithm 2” outperformed “Algorithm 1” for all subgroups differing in the consideration of smaller PTXs that limit algorithm performance. (**b**): The presence of a contralateral PTX of minor or equal size improves the algorithm-based identification of suspicious images (especially compared to “Algorithm 1”), compared with corresponding subgroups in **a**. (**c**, **d**): PTX-positive SCXRs with inserted TTs (**d**) are significantly easier to be detected compared with similar images without inlying TTs (**c**); this effect is more pronounced for “Algorithm 1.” (**a**–**d**): Areas under receiver operating curves are illustrated including the 95% confidence intervals. Subgroup definitions partially based on the pooled inclusion/exclusion of PTX size subgroups (e.g., PTX > 1 cm means the pooled consideration of PTX 1–2 cm and PTX > 2 cm). Therefore, the numbers partially do not add up. PTX-positive cases that do not meet the subgroup PTX size definitions have been excluded from ROC analysis

**Fig. 3:**
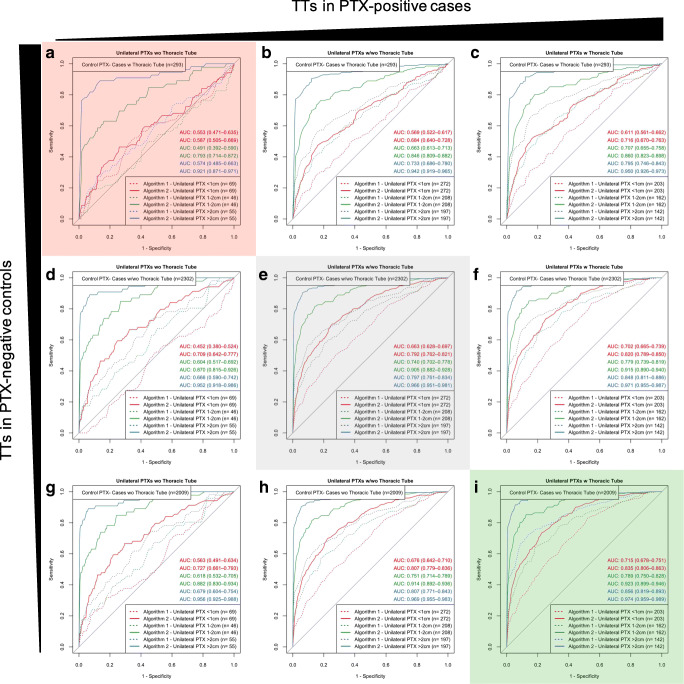
Detailed subgroup analysis (“Algorithm 1,” “Algorithm 2”) revealed thoracic tubes to be relevant confounders that can significantly bias algorithm performance. Subgroups based on PTX sizes are built for every subfigure; subfigures differ in whether PTX-positive cases and PTX-negative controls show inserted TTs. Overall performance is illustrated in the center (grayish highlighted). AUROCs for all subgroups negatively correlate with the proportion of inserted TTs in PTX-negative controls (decreasing from top to bottom). AUROCs for all subgroups positively correlate with increasing proportions of inserted TTs in PTX-positive cases (increasing from left to right). Resulting extreme scenarios are highlighted in red (algorithm discriminative performance strongly reduced) and green (best algorithm performance). Areas under receiver operating curves are illustrated including the 95% confidence intervals. PTX-positive cases that do not meet the subgroup PTX size definitions have been excluded from ROC analysis

**Fig. 4:**
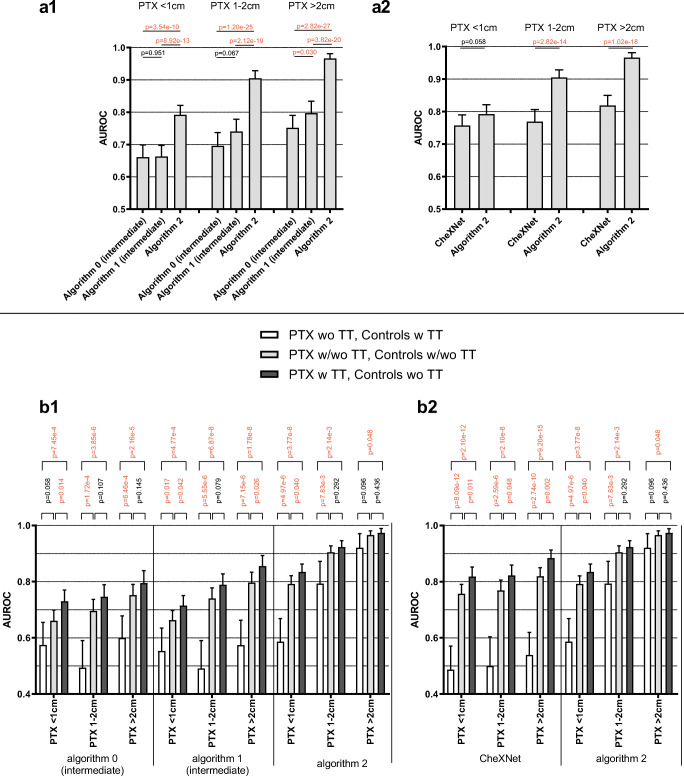
Overall performance (**a1**, **a2**) and TT-related confounding bias quantified depending on PTX size for four different algorithms (“CheXNet,” “Algorithm 0,” “Algorithm 1,” “Algorithm 2”) differing in algorithm training as described. (**a1**): In the course of algorithm development, performance could be significantly improved by considering in-image PTX annotations for algorithm training (“Algorithm 0–2"). (**a2**): The final “Algorithm 2” significantly outperformed “CheXNet” for the detection of PTXs larger than 1 cm. (**b1**, **b2**): The detection of PTX of any size by “CheXNet,” “Algorithm 0,” and “Algorithm 1” is strongly biased by inserted TTs. This confounding effect is reduced—but not eliminated—for “Algorithm 2” especially regarding PTX sizes > 2 cm with a partial loss of significance. (**a1, a2, b1, b2**): *p* values are calculated according to the DeLong method (ROC comparison); those falling below the significance threshold of *p* = 0.05 are highlighted in red. ROC subgroups analysis of “CheXNet” and “Algorithm 0” is shown in Supplemental Figure 1

**Fig. 5:**
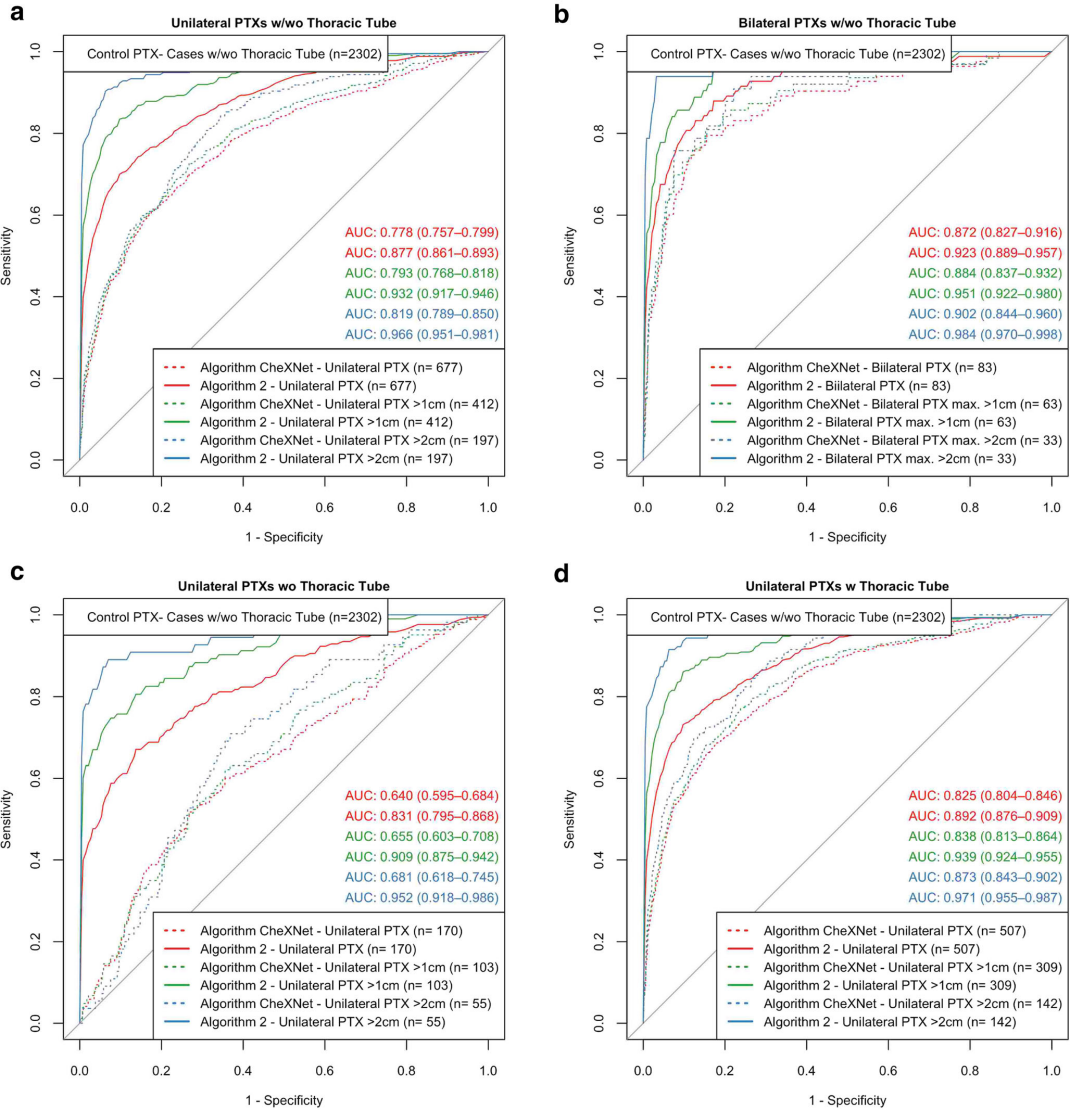
Algorithm discriminative power in pooled subgroups—comparison of the high-performing algorithms “CheXNet” and “Algorithm 2.” (**a**): “Algorithm 2” outperformed “CheXNet” for all subgroups differing in whether smaller PTXs have been also considered. (**b**): The presence of a contralateral PTX of minor or equal size improves the algorithm-based identification of suspicious images by “CheXNet” (no significant differences for “Algorithm 0”), compared with corresponding subgroups in **a**. (**c**, **d**): PTX-positive SCXRs with inserted TTs (**d**) are significantly easier to be detected by “CheXNet” compared with similar images without inlying TTs (**c**); this effect is much less pronounced for “Algorithm 2.” (**a**–**d**): Areas under receiver operating curves are illustrated including the 95% confidence intervals. Subgroup definitions partially based on the pooled inclusion/exclusion criterion of PTX size subgroups (e.g., PTX > 1 cm means the pooled consideration of PTX 1–2 cm and PTX > 2 cm); therefore, the numbers do not add up. PTX-positive cases that do not meet the subgroup PTX size definitions have been excluded from ROC analysis

Performance characterization of the algorithms by ROC analysis was based on different subgroups differing PTX size and the presence of inserted TTs in PTX-positive and/or PTX-negative CXRs. Additionally, to graphic ROC illustrations (Figs. 2, 3, and 5; Supplementary Figure 1), the most relevant resulting AUROCs will be finally compared by box plots in summarizing Fig. 4. The subgroup analysis was performed for two groups of algorithms in the same way: Analysis of “Algorithm 1” and “Algorithm 2” differing in functionality and the number of annotations considered for algorithm training is described in detail as follows (the equivalent analysis of “CheXNet” and “Algorithm 0” can be found in the Supplemental Material since similarly already introduced in a preliminary publication [16]): For unilateral PTX detection, overall performance could be quantified by AUROCs of 0.726 (0.703–0.748) for “Algorithm 1” and 0.877 (0.861–0.893) for “Algorithm 2” (see Fig. 2a). Performance of both algorithms was improved by ignoring PTX of smaller sizes. Specifically, the AUROCs increased up to 0.966 (0.951–0.981) for PTXs > 2cm. “Algorithm 2” outperformed “Algorithm 1” for all size ranges (see Fig. 2a). Subgroups of equivalent PTX sizes but additionally differing in the presence of inserted TTs in the PTX-positive images reveal TT-related confounding effects. The presence of inserted TTs in PTX-positive SCXRs facilitated their algorithm-based detection which we quantified by significantly increasing AUROCs from 0.601 (0.555–0.647) to 0.767 (0.744–0.791) for “Algorithm 1” and from 0.831 (0.795–0.868) to 0.892 (0.876–0.909) for “Algorithm 2” (see Fig. 2c, d). The influence of inserted TTs loses significance for the detection of unilateral PTXs larger than 1 cm by “Algorithm 2” which indicates that in-image PTX annotations during algorithm training might reduce possible confounding effects biasing the algorithm and resulting in the regression in performance. Please compare Fig. 2c, d and the corresponding 95% CIs. As a next step, the influence of the bilateral PTX existence was analyzed. The existence of a contralateral minor PTX increased the likelihood of an algorithm-based detection, quantified by increasing AUROCs from 0.726 (0.703–0.748) to 0.813 (0.763–0.863) for “Algorithm 1” (significant) and from 0.877 (0.861–0.893) to 0.923 (0.889–0.957) for “Algorithm 2” (not significant) (see Fig. 2a, b).

In order to quantify the confounding bias caused by inserted TTs, a detailed subgroup analysis is illustrated in Fig. 3 for “Algorithm 1” and “Algorithm 2” with subgroups built based on PTX size (separate subgroups according to size definition, see methodology) and inserted TTs in PTX-positive CXRs and inserted TTs in PTX-negative CXRs: starting in the middle (Fig. 3e), PTX-positive images (cases) as well as PTX-negative cases (controls) included images with and without inserted TTs. Shifting to the left (Fig. 3a, d, g), only those PTX-positive cases without TTs were considered; shifting to the right (Fig. 3c, f, i), only those PTX-positive cases also including inserted TTs were considered. Similarly, the upper row of the diagrams (Fig. 3a–c) corresponds to AUROCs based on PTX-negative control cases with inserted TTs and the row at the bottom (Fig. 3g–i) represents AUROCs calculated based on PTX-negative cases without any inserted TTs. The comparison of the different subgraphs revealed for all PTX-dependent subgroups AUROCs increasing from left to right (increasing number of PTX-positive cases with inserted TTs) as well as from top to bottom (decreasing number of PTX-negative controls with inserted TTs, except for PTXs < 1 cm by comparing Fig. 3 a and d supposedly caused by statistical background fluctuation for insufficient algorithm performances). This scheme results in two interesting scenarios: Discriminative power of “Algorithm 1” is completely lost for the identification of PTX-positive images (regardless of PTX size) without any inserted TTs within a group of PTX-negative control cases including inserted TTs, whereas “Algorithm 2” significantly outperformed “Algorithm 1” at least for PTX sizes larger than 1 cm (see reddish highlighted Fig. 3a). Discriminative power of both algorithms was maximally increased up to AUROCs of 0.856/0.974 (“Algorithm 1”/“Algorithm 2”) for the detection of PTX-positive images including inserted TTs within a group of PTX-negative controls without any inserted TTs (see greenish highlighted Fig. 3i).

Similar analysis was also performed by comparing the established baseline algorithms “CheXNet” and “Algorithm 0” (Supplemental Figure 1) and results of all algorithms are summarized in Fig. 4. Figure 4 a statistically compares the AUROCs for unilateral PTX detection (regardless of inserted TTs, corresponding to ROC curves in Fig. 3e and Supplemental Figure 1E): Considering the course of algorithm development (“Algorithm 0”–“Algorithm 2”), the consideration of in-image annotations in algorithm training significantly improved discriminative performance although the total number of images used for algorithm training was reduced (Fig. 4a1). In the end, the final “Algorithm 2” significantly outperformed the established benchmarking algorithm “CheXNet” for the detection of PTXs larger than 1 cm (see Fig. 4a2). Overall performances of the best competing algorithms (“Algorithm 2” vs “CheXNet”) are graphically compared in Fig. 5. Furthermore, the consideration of in-image annotations partially suppressed the confounding effects of inserted TTs (see Fig. 4b and Fig. 5c, d): The influence of TTs in PTX-positive cases/PTX-negative controls on achievable AUROCs is numerically reduced for “Algorithm 2.” This is the case especially regarding the detection of PTXs larger than 2 cm, here with a partial loss in significance. The remaining bias of Algorithm 2 in the subgroup of PTX < 1 cm (Fig. 4b) might be caused by a subgroup underrepresentation in the algorithm training: 50% of identified PTX < 1 cm cases have been designated for algorithm training but only those large enough for anatomical annotation could be finally considered. Finally, “Algorithm 2” also enables a graphic illustration of the detected findings using bounding boxes (please compare Fig. 1 c1 and c2).

## Discussion

We demonstrated that the inclusion of in-image pixel annotations in algorithm training is an effective method to significantly improve algorithm performance for PTX detection in chest radiographs. We also demonstrated that this approach of annotating the PTX shape also reduces the confounding bias that is known to be caused by inserted TTs [16].

The already established algorithm “CheXNet” achieved inferior results on our benchmarking cohort (Table 1), compared with the original publication [16, 18, 19]. This is likely supposed to be caused by our more challenging test data sets exclusively consisting of images acquired in patient’s supine position (in contrast to external test data) which yields a shift to critically ill patients with more comorbidities and a high fraction of images of limited quality since, e.g., acquired with ICU mobile devices (rotation, tilting, body parts) as well as a large proportion of TTs present even in the PTX-negative control cases [16]. In the course of algorithm development, “CheXNet” was outperformed by including in-image PTX pixel annotations in the algorithm training (“Algorithm 2”) although the total number of images considered for the algorithm training was much lower. This observation highlights the importance of training data quality that at any time might exceed the relevance of quantity. Here, training data quality could be increased by pixel annotations, which is a known method to promote machine learning [27].

In-image annotations of the dehiscent visceral pleura have been used for algorithm training not only to improve the algorithm overall performance but also to suppress the confounding bias caused by TTs that are obviously more prevalent in the PTX-positive training data (here 12.7% vs 74.9%; see Table 1) [16]. This bias is comparable with the commonly used example in the computer vision community of AI algorithms that accidentally learned to detect rails instead of trains. This was caused by an algorithm training based on images of trains, which usually run on rails. The ubiquitousness of biasing issues in AI systems has been demonstrated by several studies [28–30]. The TT-related confounding bias is briefly mentioned by other studies [31], recently quantified in detail and so far, only of TTs for algorithm training will help to further suppress this bias. However, such an approach of directly annotating confounding image features would presuppose that these confounders have been identified before (as it is the case for TTs). TTs have been the only directly investigated confounding bias, but with regard to transferability and generality of our approach and results, we have to strongly assume for any training data set and a diversity of AI applications that there might be several “hidden” confounding image features, e.g., other catheter material or comorbidities. These hidden confounders would also be addressed by directly annotating the key image region indicating the pathology that is aimed to be detected without the need to specifically know single confounders.

Limitation of our study with regard to confounding catheter material is its benchmarking single-center study design, thus allowing only the analysis based on in-hospital used TTs; therefore, other possible confounders have not been specifically addressed. Furthermore, we exclusively focused on supine chest radiographs so relative performances of the tested algorithms might differ based on images acquired in patients’ upright position. Nevertheless, exclusive focus on SCXRs provides a higher proportion of images with possible confounders, e.g., ICU patients with inserted TTs. Also, a small fraction (3.38%) of training data for Algorithm 2 were derived from the same clinical site as the test data. This fraction included PTX positive as well as PTX negative (each with/without inserted TTs). Keeping this in mind, we therefore assume that PTX-detecting algorithm performance within our single-center benchmarking cohort should not be biased to a major extent. Also, good coverage of sites, vendors, and image flavors within our training dataset allows for good performance generalization. Another limitation is related to the annotation quality; specifically, based on annotations performed by medical students (supervised, well trained as described), we must assume a small amount of annotation error yielding marginal overlaps within subgroups and the assessment of questionable small PTXs as positive or negative based on clinical plausibility supposedly yields an unavoidable slight blurring of our reference standard. It should also be kept in mind that especially those SCXRs with inserted TTs might be radiologically classified as “false” negative for PTX in case of a residual PTX which is radiographically not detectable (neither for radiologists and probably nor for algorithms). Also, inter-reader variability might affect the subgroup compositions based on measured PTX sizes, especially close to the subgroup boundaries; however, these errors can be assumed to be bidirectional uniform, thus not yielding any systematic preference for any subgroup. PTX size measurements have also been demonstrated to vary only to a limited extent with regard to intra- and inter-reader variability [32].

In conclusion, we used the AI-based PTX detection as an example to demonstrate that in-image pathology pixel annotations are an effective method to significantly improve the training of pathology-detecting AI algorithms. Through extensive experiments, we demonstrated that one can achieve a boost in algorithm performance and significantly reduce the influence of confounders that can be identified in detailed test data subgroup analysis. These approaches are crucial to avoid diagnostic AI algorithms that unknowingly underperform in specific patient subgroups and therefore would have the risk of patient hazard in clinical routine. In this context, we established a clinically relevant and radiologically annotated benchmarking cohort that can also be used for further evaluation of PTX-detecting AI algorithms.

## Supplementary information

ESM 1 (DOCX 748 kb)

## Abbreviations

95% CI: 95% confidence interval
AI: Artificial intelligence
AUROC: Area under receiver operating characteristics
CXR: Chest X-ray
ICU: Intensive care unit
NLP: Natural language processing
PTX: Pneumothorax
SCXR: Supine chest X-ray
TT: Thoracic tube

## Footnotes

1 In case of a possible or suspected PTX from the clinical point of view (e.g., preceding intrathoracic catheter insertion/intervention or a known PTX in previous examinations), these images have been assessed as positive for PTX (since any algorithm should also raise suspicion here). Contrarily, images with PTX mimickers have been considered negative given that there is no reason for PTX suspicion from the clinical point of view.

2 A total of 43,457 training cases of patients older than 18 from four different clinical institutions (three in Europe, one in the USA) have been identified, among them 2537 chest radiographs acquired in patient’s supine position (the rest PA/erect). Images have been acquired with a variety of vendors (e.g., Siemens, Philips, GE, AGFA, Fujifilm, Kodak, Carestream, Canon, Varian, Konica Minolta, Swissray). PTX-positive cases were annotated by expert radiologists using an internal tool (similar to ITKSnap) to mark the contour of the pleural line.

## Declarations

### Guarantor

Dr. Johannes Rückel, B.Sc.

### Statistics and biometry

No complex statistical methods were necessary for this paper. Several authors have significant expertise for the applied methods.

### Study subjects or cohort overlap

Study subjects have been partially reported:

Johannes Rueckel, Lena Trappmann, Balthasar Schachtner, et al: Impact of Confounding Thoracic Tubes and Pleural Dehiscence Extent on Artificial Intelligence Pneumothorax Detection in Chest Radiographs. Investigative Radiology 2020

### Methodology

• retrospective

• diagnostic study

• performed at one institution
