# Supplementary material for: Pneumothorax detection in chest radiographs: optimizing artificial intelligence system for accuracy and confounding bias reduction using in-image annotations in algorithm training
Source: Eur Radiol. 2021 Mar 27;31(10):7888–900. doi: 10.1007/s00330-021-07833-w (PMC8452588; doi:10.1007/s00330-021-07833-w)
Supplement: Supplementary file 1 — (DOCX 748 kb) [file 330_2021_7833_MOESM1_ESM.docx]

**ELECTRONIC SUPPLEMENTARY MATERIAL**

**Algorithm Descriptions**

**AI Algorithm 0**

The architecture of the AI Algorithm 0, proposed by Guendel et al. (1), is illustrated in Figure 2. It comprises of three main parts:

1. **Image resampling component:** The input image is resampled to a fixed size of 1024 x 1024 pixels using bilinear interpolation and is replicated into three channels for compatibility with a pre-trained RGB model (see the part DL model). The 3-channel RGB input is then processed by two convolutional layers each with a filter size of 3 x 3 and a kernel shift (i.e., stride) equal to 2. The two convolutional layers reduce the image size, modeling the standard Gaussian down-sampling operation.
2. **DL model:** A pre-trained densely connected convolutional neural network (DenseNet) with 121 layers is used, which was initially proposed by Hua et al. (2) and is publicly available, see [https://github.com/liuzhuang13/DenseNet]. The fundamental idea of DenseNet model is to alleviate the vanishing gradient problem and to reduce the number of network parameters by exploiting fully connected blocks with direct links from each layer to all preceding layers.
3. **Labels and cost function:** The network illustrated in supplemental Figure 1 is trained using the ChestX-Ray 14 and PLCO datasets (3, 4). As the label definitions cannot be guaranteed to be identical across the two datasets, one network output is associated with each dataset-dependent pathology. Furthermore, spatial information of certain findings, not including PTX, is provided by PLCO and considered by including a set of additional image-level labels in training. The training is performed by using the binary cross entropy loss function weighted with the label frequency within each batch (size of 128) to account for the positive and negative class imbalance.

**
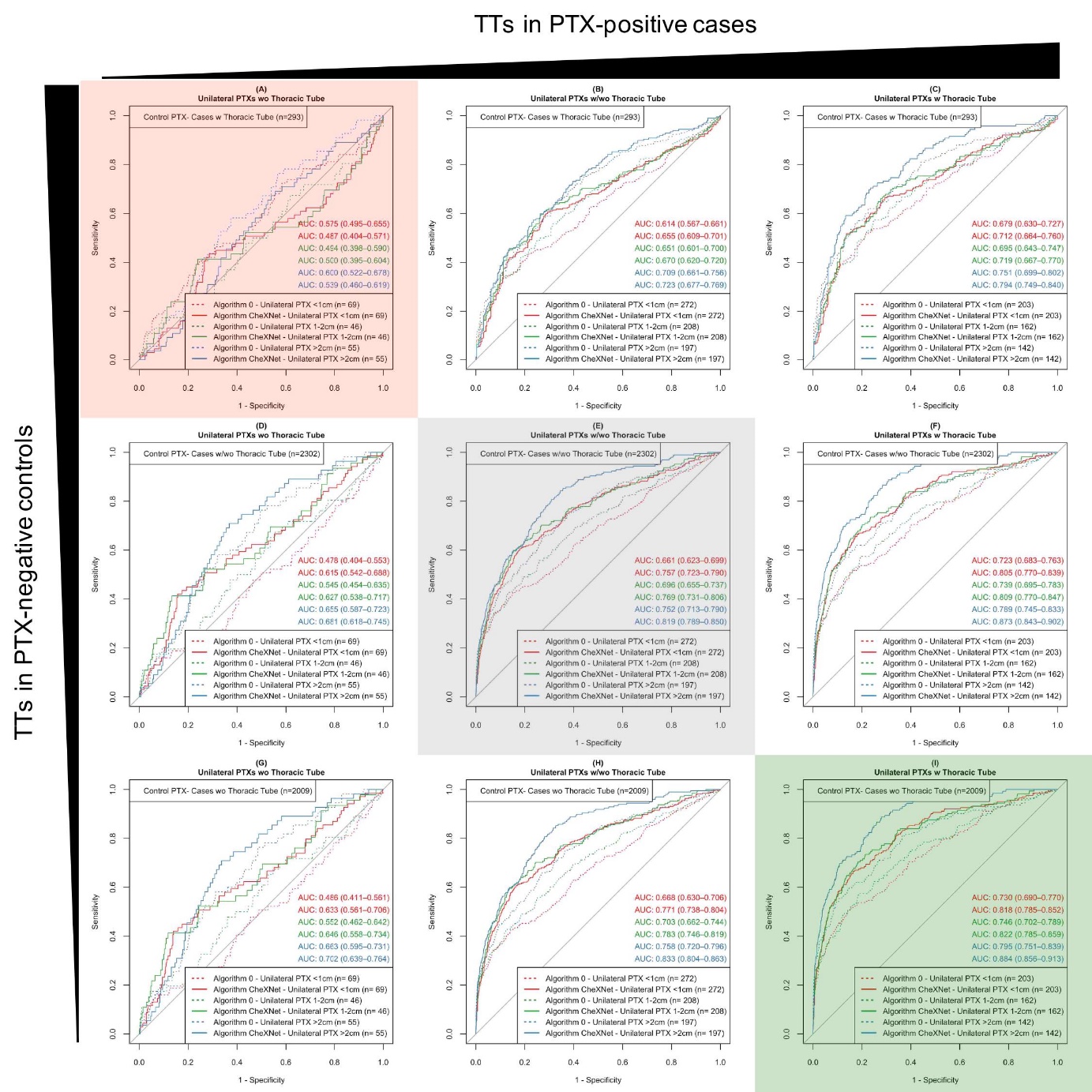
**

**Supplemental Figure 1: Detailed subgroup analysis revealed thoracic tubes to be relevant confounders that significantly bias algorithm performance (“CheXNet”, “algorithm 0”).** Subgroups based on PTX size are built for every subfigure; subfigures differ in the proportion of inserted TTs in PTX-positive cases and PTX-negative controls. Overall performance is illustrated in the center (grayish highlighted). AUROCs negatively correlate with the proportion of inserted TTs in PTX-negative controls (decreasing from top to bottom). AUROCs for all subgroups positively correlate with increasing proportions of inserted TTs in PTX-positive cases (increasing from left to right). Resulting extreme scenarios are highlighted in red (algorithm discriminative performance strongly reduced) and green (best algorithm performance). Areas under receiver operating curves are illustrated including the 95% confidence intervals. The graph basically corresponds to Rueckel et al. [16]; but in this case only adult patients of the testing cohort have been analyzed which enables the statistical comparison with “algorithm 1” and “algorithm 2” (see figure 4). PTX positive cases that do not meet the subgroup PTX size definitions have been excluded from ROC analysis.


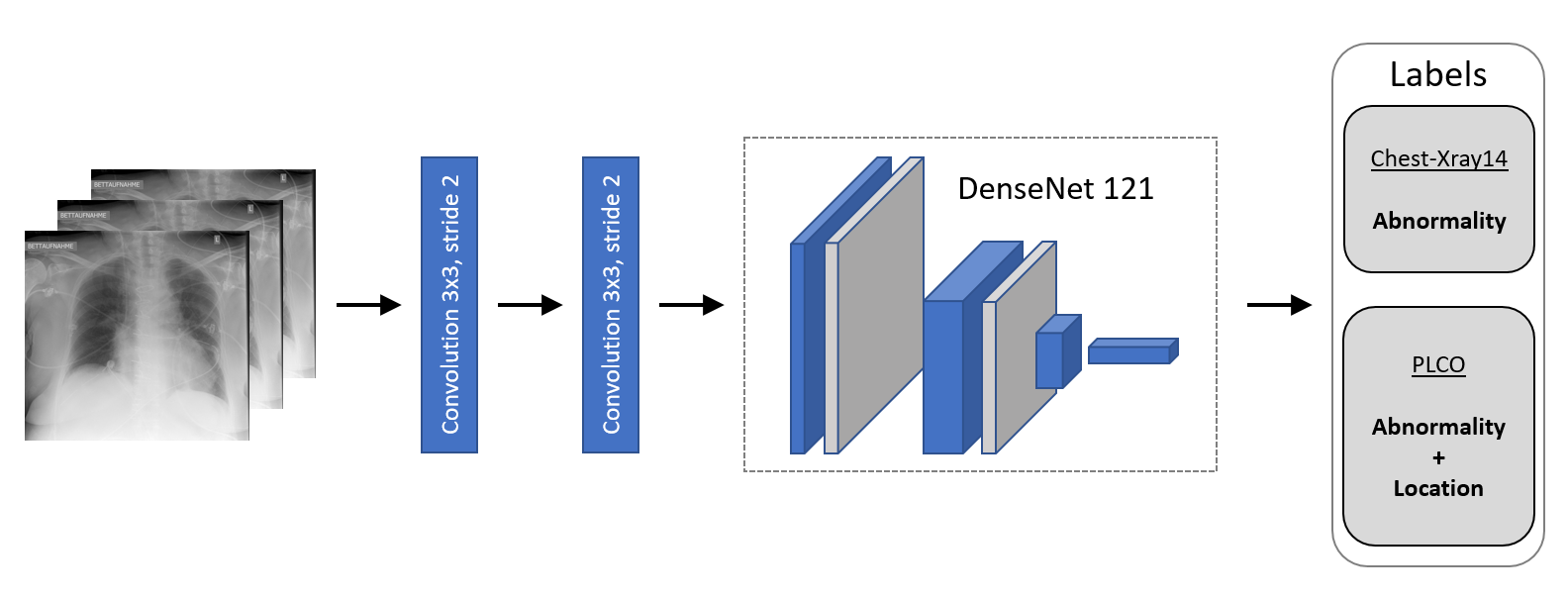


**Supplemental Figure 2:** Architecture of the AI Algorithm 0 based on a locate-aware dense network.

**AI Algorithms 1 and 2**

The architecture used by both AI Algorithms 1 and 2 is illustrated in supplemental Figure 3. The architecture comprises of two main components, namely, dehiscent visceral pleura localization module and pneumothorax classification module.

1. **Localization module:** In order to constrain the classification decision, our approach begins by the localization of pneumothoraxes. Specifically, the input image is resampled to a fixed size (512x512 pixels) and processed by an encoder-decoder convolutional architecture to localize dehiscent visceral pleura by estimating its contours as a binary image mask.
2. **Classification module:** Features from an intermediate layer of the decoder sub-network of the localization module are concatenated with the original input radiograph and subsequently processed by the classification module to obtain an image-level probability score for pneumothorax. The architecture of the classification module is inspired by the the DenseNet architecture proposed by Hua et al. (2). In theory, one can optimize both modules independently or jointly. In our experiments, we first conducted the pretraining of the localization model separately followed by the joint training of the classification and the localization models. The training is jointly driven by a segmentation loss (an adaptive DICE loss) and classification loss (binary cross entropy).


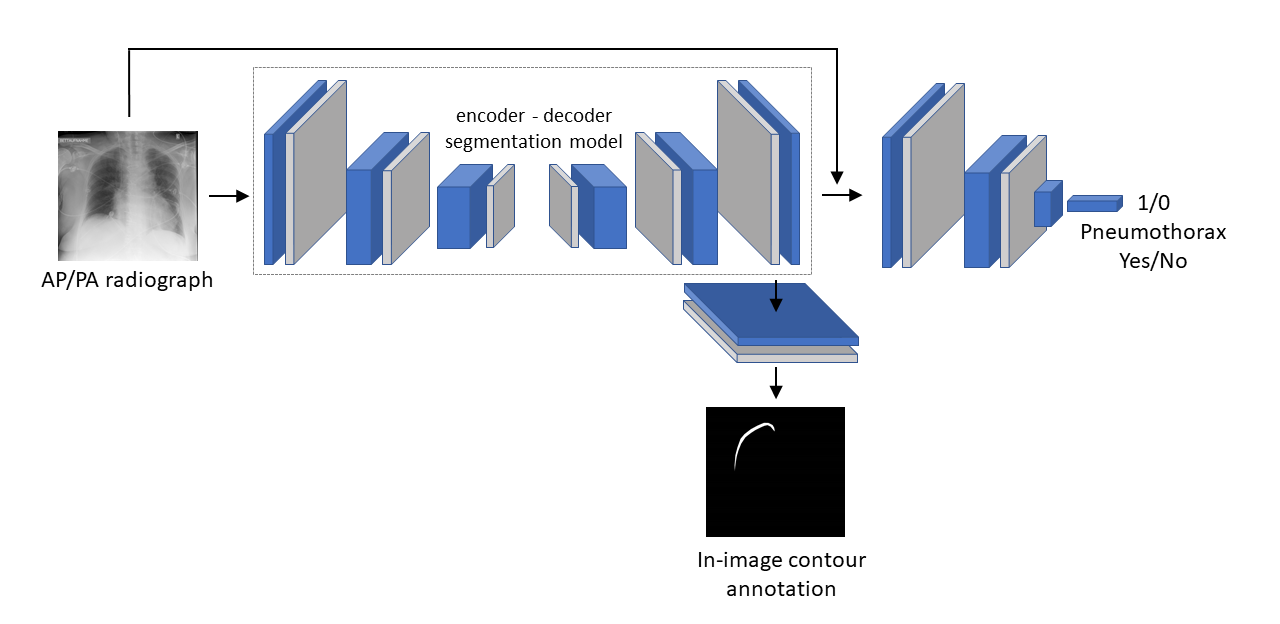


**Supplemental Figure 3:** Architecture of the system behind Algorithms 1 and 2.

**References:**

1. Guendel S, Grbic S, Georgescu B, et al.: Learning to recognize Abnormalities in Chest X-Rays with Location-Aware Dense Networks [Internet]. arXiv:180304565 [cs] 2018; [cited 2020 Feb 19] Available from: http://arxiv.org/abs/1803.04565

2. Huang G, Liu Z, van der Maaten L, et al.: Densely Connected Convolutional Networks [Internet]. *arXiv:160806993 [cs]* 2018; [cited 2020 Feb 19] Available from: http://arxiv.org/abs/1608.06993

3. Wang X, Peng Y, Lu L, et al.: ChestX-ray8: Hospital-scale Chest X-ray Database and Benchmarks on Weakly-Supervised Classification and Localization of Common Thorax Diseases [Internet]. *arXiv:170502315 [cs]* 2017; [cited 2018 Oct 29] Available from: http://arxiv.org/abs/1705.02315

4. Gohagan JK, Prorok PC, Hayes RB, et al.: The Prostate, Lung, Colorectal and Ovarian (PLCO) Cancer Screening Trial of the National Cancer Institute: history, organization, and status. *Control Clin Trials* 2000; 21:251S-272S
